# Exploring targeting peptide-shell interactions in encapsulin nanocompartments

**DOI:** 10.1038/s41598-021-84329-z

**Published:** 2021-03-02

**Authors:** Wiggert J. Altenburg, Nathan Rollins, Pamela A. Silver, Tobias W. Giessen

**Affiliations:** 1 Department of Systems Biology, Harvard Medical School, Boston, MA 02115 USA; 2 Wyss Institute for Biologically Inspired Engineering at Harvard, Boston, MA 02115 USA; 3 Department of Biomedical Engineering, University of Michigan, Ann Arbor, MI 48109 USA; 4 Department of Biological Chemistry, University of Michigan Medical School, Ann Arbor, MI 48109 USA

**Keywords:** Molecular engineering, Nanobiotechnology, Biotechnology, Proteins, Protein analysis, Protein design, Protein structure predictions

## Abstract

Encapsulins are recently discovered protein compartments able to specifically encapsulate cargo proteins in vivo. Encapsulation is dependent on C-terminal targeting peptides (TPs). Here, we characterize and engineer TP-shell interactions in the *Thermotoga maritima* and *Myxococcus xanthus* encapsulin systems. Using force-field modeling and particle fluorescence measurements we show that TPs vary in native specificity and binding strength, and that TP-shell interactions are determined by hydrophobic and ionic interactions as well as TP flexibility. We design a set of TPs with a variety of predicted binding strengths and experimentally characterize these designs. This yields a set of TPs with novel binding characteristics representing a potentially useful toolbox for future nanoreactor engineering aimed at controlling cargo loading efficiency and the relative stoichiometry of multiple concurrently loaded cargo proteins.

## Introduction

Compartmentalization is a key component of both eukaryotic and prokaryotic metabolism^1–3^. While the membrane-bound organelles of eukaryotes have been studied in detail for decades, prokaryotic protein-based organelles have only recently attracted increased attention^4^. Self-assembling protein compartments can be found in a variety of contexts, including virus-like capsids^5^, ferritins^6^ and bacterial microcompartments (BMCs)^7,8^. Nature uses protein compartments as molecular containers protecting nucleic acid cargo, as iron-storage compartments (ferritins) and as nanoreactors involved in carbon fixation and carbon source utilization (BMCs)^9^.

**Figure 1:**
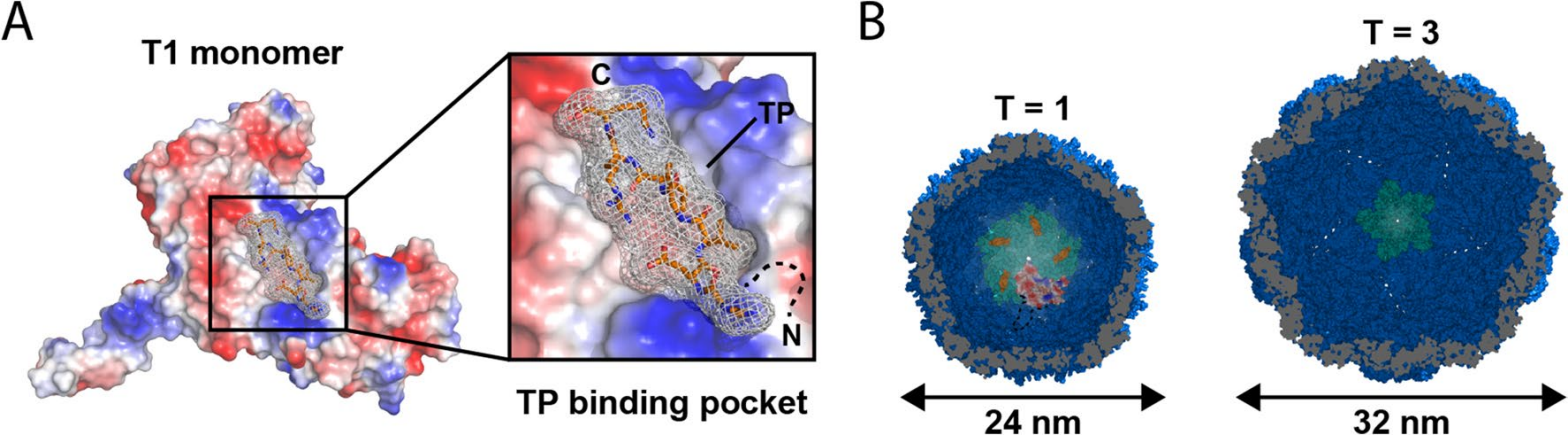
Encapsulin structure and cargo loading. (**A**) Electrostatic surface representation of a *T. maritima* T1 encapsulin protomer (PDB ID: 3DKT) in complex with its native targeting peptide viewed from the interior side of the assembled shell. The inset shows the targeting peptide binding pocket. (**B**) Cut-away views of T1 (*T. maritima*) and T3 (*M. xanthus*, PDB ID: 4PT2) encapsulins viewed along the fivefold symmetry axis. A single T1 protomer (electrostatic surface) and five targeting peptides (orange) are highlighted. The protomers forming a pentameric facet in the viewing direction are shown in green.

The most recent addition to the list of protein-based compartments are the so far identified two families of encapsulin nanocompartments found in a wide variety of bacteria and archaea^10–12^. Encapsulins are involved in oxidative stress resistance^13–15^ and iron mineralization^16^ and have been implicated in many other cellular processes^17^. We focus on family 1 encapsulins which assemble from a single type of shell protein into compartments between 24 and 42 nm in diameter with either T1, T3 or T4 icosahedral symmetry^15,16,18,19^. These encapsulins are of particular interest because a modular mechanism of cargo loading has been identified^19,20^. In family 1 encapsulins, encapsulation is mediated by short peptide sequences called targeting peptides (TPs) usually found at the C-terminus of dedicated cargo proteins (Fig. 1A).

In the laboratory, protein compartments have been used as molecular containers for diverse cargo molecules, including nucleic acids, proteins, inorganics and small molecules^9^. These systems have been employed as delivery devices, bioimaging agents, nanoreactors and artificial metabolons^21–24^. Encapsulins, possessing a dedicated cargo targeting system, seem ideally positioned for a variety of engineering applications where macromolecules need to be co-localized or sequestered inside living cells^25^. Although, heterologous cargo has been successfully targeted to the interior of encapsulins, a lack of understanding of cargo encapsulation has hampered the design and engineering of more advanced multi-component nanoreactors and artificial organelles.

Here, we characterize TP-shell interactions in the *T. maritima* (24 nm, 60 subunits)^19^ and *M. xanthus* (32 nm, 180 subunits)^25^ encapsulin systems (Fig. 1B). We then computationally design and experimentally characterize a set of TPs with varying relative binding strengths, thus laying the foundation for future advanced nanoreactor engineering.

## Results and discussion

### Computational analysis of TP-shell interactions

**Figure 2:**
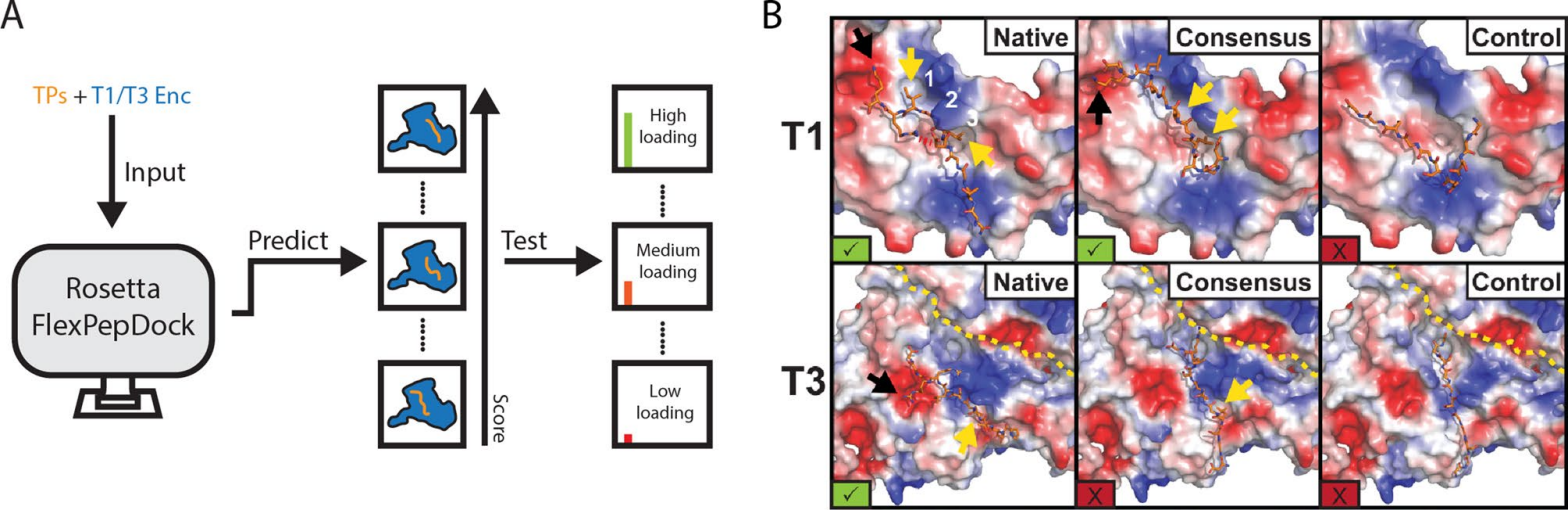
Flexible docking predicts shell-targeting peptide interactions and relative binding strength. (**A**) Schematic of combined computational and experimental workflow. (**B**) For some peptides, force-field docking found top-scoring TP conformations with sufficient ionic (black arrow) and hydrophobic (yellow arrow) interactions to bind encapsulin (green check mark). For others (red X), insufficient interactions were found to expect binding. The binding surface of T3 crosses two subunits (protomers separated by yellow dash).

Computational modeling indicates that TP binding is mediated by key hydrophobic and ionic interactions that depend on the relative positioning of anchor residues and TP flexibility. Rosetta FlexPepDock^26,27^ was used to gain a more detailed understanding of TP-shell protein interactions and to develop a workflow for the prediction of relative TP binding strengths that could be used to generate new customized TPs (Fig. 2A). For our calculations, we used native TPs (T1: NTGGDLGIRK, T3: PLTVGSLRRGG), consensus TPs found in T1 and T3 encapsulin systems (T1: DGSLGIGSLKG, T3: DGSLGIGSLRG) and a GGS control TP (GGSGGSGGSGG) as inputs. First, all calculated interactions of TPs and binding sites (15,000 per TP) resulting from a FlexPepDock ab initio step followed by a refinement protocol were sorted based on their scores. The top 500 by lowest score were taken and clustered with an RMSD of 3 Å to differentiate between different binding modes. The lowest energy structure in the largest cluster was chosen to be the representative interaction. To calibrate our modeling approach, we used the co-crystal structure of the *T. maritima* encapsulin (T1) and its native TP (Fig. 1A). The modeled structure for the native T1 TP-encapsulin interaction is in good agreement with the experimental co-crystal structure with a peptide backbone root-mean-square deviation (RMSD) of 2.2 Å (Supplementary Information Fig. S1).

It should be kept in mind that our analysis is solely focused on the interaction of a single TP with its binding site and does not consider concurrent TP binding events at neighboring sites. With the goal of using TPs as modular targeting tags in the future, we do not consider the influence of the native cargo protein domain on TP binding which is rationalized by the fact that TPs are always connected to the native cargo domain via 10 to 20 residue flexible linkers which will decrease the influence of cargo domain identity on TP-shell interactions. Thus, our computational analysis purely focused on TP-shell binding represent an approximation of cargo binding and loading that does not consider multiple TP binding events and the influence of a cargo domain connected to the TP. Both of these effects are likely to influence the configurational entropy of TPs in the context of cargo loading and are likely to decrease TP binding, especially for short linkers, bulky cargo domains, and high cargo concentrations.

In our modeling results, the native TPs showed the best scores in both the T1 and T3 systems (Supplementary Information Table S1). The T1 consensus TP had a similar but slightly lower score compared with the native T1 TP while the control TP had the lowest score in the T1 system calculations (Fig. 2B). The T3 consensus and control TPs had comparable and low scores, predicting that the chosen T3 consensus TP will likely show only low levels of cargo loading.

The T1 encapsulin protein structure contains a hydrophobic pocket consisting of 3 grooves (Fig. 2B, white numbering) which serve as the main anchor points for both the native and consensus TPs. In both cases the interaction is governed by two hydrophobic residues separated by a glycine (LGI). The glycine residue serves as a flexible hinge allowing the two hydrophobic residues to adopt optimal conformations for interacting with the hydrophobic pocket. In the native T1 TP, the isoleucine interacts with the third groove of the pocket while the T1 consensus TP adapts a conformation where the isoleucine is able to interact with the second groove (Fig. 2B, yellow arrows), more similar to the binding mode found in the co-crystal (Fig. 1A). In addition, the lysine residues at or close to the C-terminus, present in both the native and consensus T1 TPs, interact with a negative surface patch of the T1 encapsulin (Fig. 2B, black arrows). The modeled native T1 TP shows an intra-peptide interaction between the penultimate arginine and the glutamate residue (Fig. 2B), very similar to the conformation observed in the T1 co-crystal structure again validating our modeling approach (Fig. 1A). This missing interaction as well as the presence of an additional leucine residue showing an unfavorable conformation are likely the reasons for the slightly lower score observed for the T1 consensus TP. The GGS control TP is not able to bind in the hydrophobic pocket resulting in an overall lower score (Supplementary Information Table S1).

Because no structural information about the interaction of a TP with a T3 encapsulin (*M. xanthus*) exists and the fact that the binding surface is likely located close to a subunit interface, we decided to include two subunits of the T3 encapsulin protomer in the modeling of the T3 system. The T3 modeling results suggest that binding of the native TP is primarily mediated by the hydrophobic interaction of a valine residue and the ionic interaction of an arginine placed five residues apart (Fig. 2B, yellow and black arrows, respectively). The arginine tightly interacts with a negatively charged surface patch forming 3 hydrogen bonds. The interacting motif in the native T3 TP is VxxxxR with the residues between V and R (GSLR) allowing enough flexibility for optimal binding and the serine residue interacting with a positive surface patch. The GSL motif is widely conserved^19^ in TPs highlighting its importance for optimally positioning the interacting residues. In the T3 consensus TP, the defining interaction is the penultimate arginine residue. The spacing of hydrophobic residues does not allow for a V/L/IxxxxR motif which likely contributes to the low score observed for the T3 consensus TP. Again, the GGS control TP is not able to form any of the core interactions observed for the native T3 TP and shows a similarly low score as the T3 consensus TP (Supplementary Information Table S1).

### Experimental characterization of cargo loading

**Figure 3:**
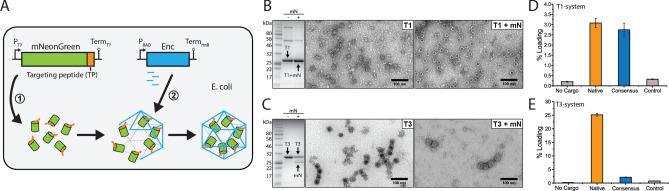
Bulk characterization of encapsulin cargo loading. (**A**) Experimental plan outlining the production and encapsulation of heterologous fluorescent cargo to test targeting peptide strength. (**B**) and (**C**) SDS-PAGE gels and negatively stained transmission electron micrographs of purified encapsulin particles using native TPs. (**D**) and (**E**) Bulk fluorescence measured for the indicated systems and targeting peptides. Error bars represent standard deviations resulting from three independent replicates.

To test the predictions made by our modeling, an experimental system was designed where mNeonGreen was C-terminally fused with different TPs (mNeonGreenTP) and fluorescence would serve as a read-out of in vivo cargo loading in purified encapsulin particles. The *T. maritima* (T1 system) and *M. xanthus* (T3 system) encapsulin proteins were used as the encapsulation component of the system. mNeonGreenTP and encapsulin constructs were designed to be independently inducible. In all experiments, cargo would be induced first and allowed to accumulate in *Escherichia. coli* before starting encapsulin production (Fig. 3A). This setup will prevent other factors like translational coupling from interfering in the determination of relative TP strength as much as possible.

We established an expression and purification system for T1 and T3 encapsulins and their respective cargo (Supplementary Information Figs. S2 and S3). *E. coli* strains encoding mNeonGreenTP variants and encapsulins on two independently inducible plasmids were constructed. After expression of mNeonGreenTP for 6 h, encapsulin expression was initiated. Nanocompartments were purified via a three-step protocol, starting with polyethylene glycol precipitation, and followed by size-exclusion and anion exchange chromatography. We were able to purify readily assembled nanocompartments from all strains as indicated by transmission electron microscopy (Fig. 3B,C and Supporting Information Figs. S4 and S5). SDS-PAGE analysis and in-gel tryptic digest mass spectrometry showed that mNeonGreenTP cargo could be co-purified with encapsulin particles for both the T1 and T3 systems. In case of the T1 system, mNeonGreenTP and the T1 encapsulin protein had nearly identical molecular weights and could not be resolved via SDS-PAGE whereas two clear bands could be seen for purified T3 particles. Further, successful co-purification of assembled encapsulins and heterologous fluorescent cargo was additionally confirmed using native PAGE and in-gel fluorescence analysis of high molecular weight bands (Supplementary Information Figs. S6 and S7).

Using bulk plate-reader fluorescence measurements, the loading of T1 and T3 encapsulins was determined. We defined loading percentage as the calculated percentage of occupied TP binding sites based on the total number of available binding sites (T1: 60 binding sites, T3: 180 binding sites). This is necessary because limited information exists about native levels of cargo loading while loading would also ultimately depend on cargo protein size for differently sized systems. In case of the T1 system, similar cargo loading percentages of 3% (ca. 2 cargo proteins per shell) were observed for both the native and consensus TPs while the control TP showed only background levels of fluorescence (Fig. 3D). This is in good agreement with our modeling results where the native and consensus TPs showed similar scores, both distinctly higher than the control. Cargo loading in the T3 system was 25% (ca. 45 cargo proteins per shell) for the native TP while the consensus and control TPs showed only background levels of loading (Fig. 3E). This finding is again in agreement with our modeling results which predicted high loading for the native T3 TP and low levels of loading for the T3 consensus TP. The difference in loading percentage between the T1 and T3 systems may be a result of the non-native two plasmid expression system and the presumably faster assembly of the 60 subunit T1 encapsulin compared with the 180 subunit T3 system. Native encapsulin systems are always arranged in an operon structure (cargo followed by encapsulin protein) controlled by a single promoter. The higher levels of loading observed for some native encapsulin systems when arranged in an operon structure point towards strong translational coupling effects^28^. However, to decouple cargo loading from these effects and characterize TP targeting strength we decided to utilize a two-plasmid setup. Another likely factor that decreases cargo-loading efficiency is the use of a non-native cargo protein (mNeonGreenTP) which is not evolutionarily optimized for encapsulation and thus may result in pronounced negative steric effects during cargo loading and shell assembly, especially for the smaller T1 system.

**Figure 4:**
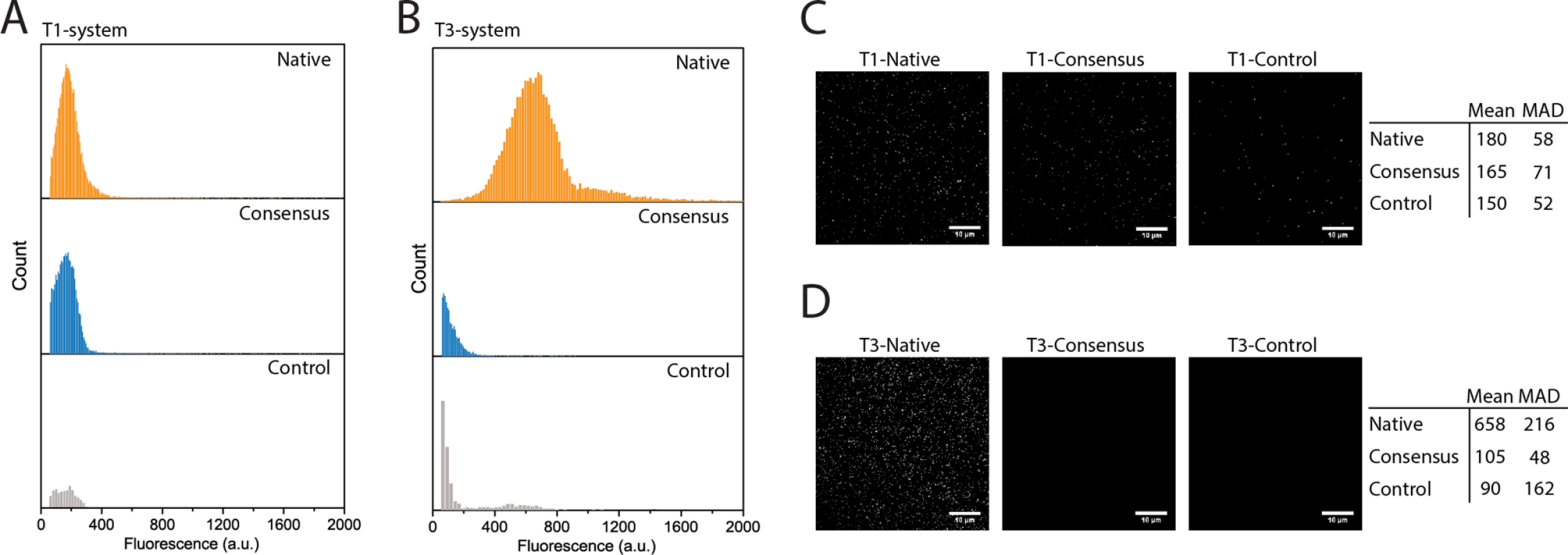
Single particle characterization of encapsulin cargo loading. (**A**) and (**B**) Histograms of single particle fluorescence measurements for T1 and T3 systems with different targeting peptides. (**C**) and (**D**) Example single particle fluorescence images of immobilized mNeonGreen-loaded encapsulins. The native T3 system showed greater fluorescence per particle than the T1, as expected due to the larger size and subunit number of T3. The T1 consensus showed slightly less loading than native, whereas the T3 consensus peptide showed greatly diminished loading only slightly stronger than GGS controls. Scalebar: 10 μm. Mean: mean fluorescence of measured spots. MAD: mean absolute deviation, see Eq. (3).

To gain a deeper understanding of cargo loading distributions, single particle florescence imaging was used. Whereas the T1 system showed consistent unimodal distributions of cargo loading for all TPs (Fig. 4A,C), the native T3 TP indicated bimodality (Fig. 4B,D). This can be explained by assuming a high loading rate of T3 encapsulins shortly after induction of encapsulin protein production because of an abundance of cargo. Due to the declining availability of cargo protein, continuing encapsulin production will then lead to lower loading as expression continues. In contrast, the overall low loading observed for the T1 system guarantees high cargo availability even after prolonged encapsulin production.

### Mutant TPs with novel targeting strengths

**Figure 5:**
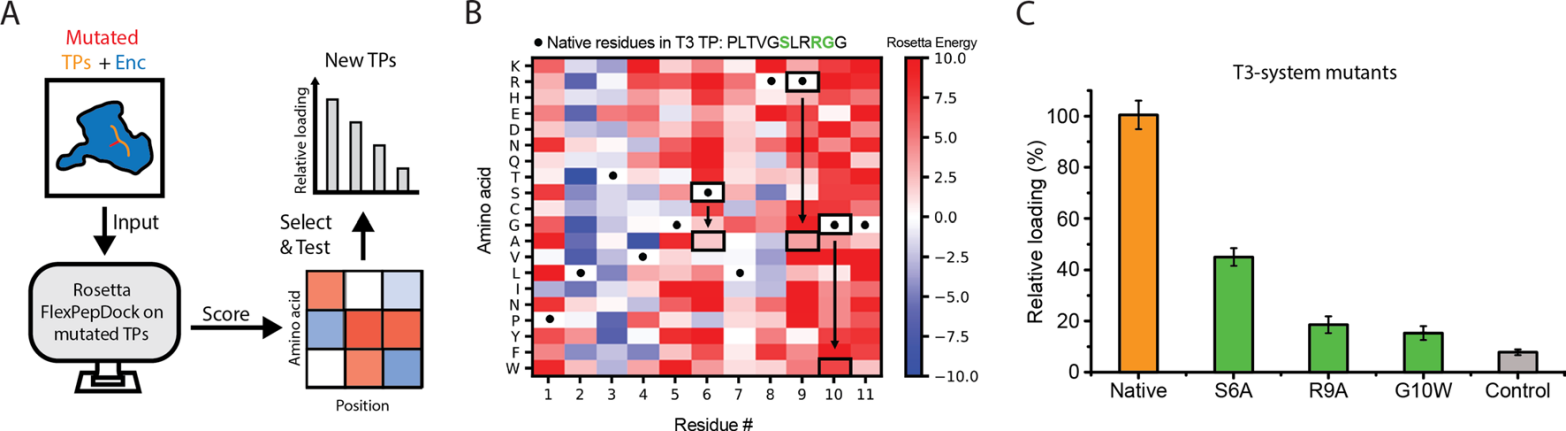
Design and characterization of novel targeting peptide mutants. (**A**) Workflow outlining the design and testing of mutant targeting peptides. (**B**) Heat map of computationally generated point mutations within the *M. xanthus* T3 targeting peptide. The color gradient represents the Rosetta Energy score (blue: improved binding, red: worse binding). Native sequence residues are indicated by asterisks. The mutants selected for experimental testing are shown with black arrows pointing from the native residues (asterisk) to the mutant residues. (**C**) Bulk fluorescence measurements of mutant targeting peptides. Error bars represent standard deviations resulting from three independent replicates. Fluorescence measurements of the native and control TP for the T3 system are shown for comparison.

We now turned to applying our modeling approach to the generation of completely novel TPs with a range of distinct targeting strengths (Fig. 5A). We focused on the T3 system due to the T3 encapsulin’s larger size and the systems higher overall loading efficiency in a multi plasmid context. Both properties would make the T3 system a more versatile platform for nanoreactor engineering.

We carried out a comprehensive in silico point mutation screen of the native 11-residue T3 TP (Supplementary Information Table S2). Each residue was substituted for all 20 standard amino acids yielding 220 unique mutant TPs (Fig. 5B). To model mutant TP targeting strength, a FlexPepDock refinement protocol was employed. Based on our computational analysis, the majority of mutations will have negative effects on binding strength. Especially the last three TP residues (RGG, position 9 to 11) seem to influence binding, since most mutations result in positive energy scores and thus negatively impact binding (Fig. 5B). This likely results from disturbing the anchoring interaction of the arginine residue at position 9 which is important for TP-shell interactions (Fig. 2B). Further, mutations at positions 5 to 7 (GSL) also result in primarily worse binding scores. The GSL motif is likely important for allowing enough TP flexibility for shape similarity matching while the serine at position 6 additionally forms a hydrogen bond within the TP binding site. Mutating residues 2 to 4 (LTV) results in primarily improved binding characteristics, as calculated in silico.

To investigate the validity of our computational predictions, we decided to produce and experimentally characterize three mutant TPs. Here, we focused on mutations that would decrease targeting strength. The following mutant TPs were characterized: S6A, R9A and G10W. After purification of encapsulins carrying mNeonGreen fused to mutant TPs, bulk fluorescence measurements were carried out. The results indicate that as expected, all mutants result in diminished cargo loading (Fig. 5C). Loading roughly follows the expected trend based on computationally calculated scores with the S6A mutant showing the least and G10W mutant the strongest decrease in cargo loading.

In conclusion, we carried out a focused characterization of TP-shell interactions in T1 and T3 encapsulin systems and developed a computational approach with which to generate novel TPs with modulated relative targeting strengths. Although only an approximation of the complexities of cargo loading as outlined above, the characterization of TP-shell binding is likely a useful piece of information for designing novel TPs. Expanding this approach will allow advanced nanoreactor engineering in the future where TPs of distinct relative targeting strengths can be mixed and matched to achieve defined in vivo loading of multiple cargo proteins, both in terms of absolute amount and relative cargo stoichiometry. This ability is a prerequisite to gain proper control over encapsulin systems in vivo with the ultimate goal of creating customized compartmentalized spaces inside cells whose properties can be controlled at the molecular level to address specific challenges in metabolic engineering, biocatalysis and bionanotechnology.

## Methods

### Molecular biology techniques

All DNA was ordered through IDT DNA technologies (IDT). Constructs were optimized using the IDT Codon Optimization Tool for *E. coli* with the amino acid sequence as input and ordered as gBlock Gene Fragments (gBlocks). The vectors pCDF-Duet1 (mNeonGreen-TP) and pBAD/HisA (encapsulin constructs) were used for all cloning procedures digested using NdeI and PacI or NcoI and XhoI, respectively. All constructs were assembled using Gibson Assembly; Gibson Assembly Master Mix was obtained from New England BioLabs (NEB). The respective gBlocks contained 20 bp overlaps for direct assembly into the digested vectors. Before expression, all constructs were verified using Sanger sequencing (GENEWIZ).

### Protein expression and purification

Constructs were (co-)transformed into One shot BL21 (DE3) Star cells (ThermoFisher), 15 ng of each plasmid was used. Expressions were carried out using lysogeny broth (LB) supplemented with ampicillin (100 μg ml^−1^), spectinomycin (100 μg ml^−1^) or both. 50 mL of LB was inoculated using an overnight culture grown at 37 °C and 200 rpm to an optical density (OD_600_) of 0.6 and then induced by isopropyl β-d-1-thiogalactoyranoside (IPTG), 0.1 mM final concentration, and/or l-arabinose (0.2% w/v final concentration) for 6 h at 20 °C and 200 rpm. Media containing IPTG was removed using centrifugation (4000 rpm, 10 min, 4 °C) to terminate induction of cargo expression. Cells were diluted to an OD_600_ of 0.6 in 100 mL of fresh LB supplemented with antibiotics and induced with l-arabinose (0.2% w/v final concentration) and expressed for 18 h at 20 °C and 200 rpm. Cells were collected using centrifugation (4000 rpm, 10 min, 4 °C) and the pellets were used directly, or flash frozen in N_2_ and stored at − 20 °C.

Cell pellets were suspended in 5 mL of PBS (pH 7.4). Lysozyme (Sigma-Aldrich) and DNAseI (Sigma-Aldrich) were added and the suspension was lysed using a 550 Sonic Dismembrator (FisherScientific). Power level 3.3 was used with a pulse time of 8 s and 10 s interval time. The suspension was centrifuged for 15 min, 8000 rpm and at 4 °C. To the lysate, 0.1 g NaCl and 0.3 g (T3 encapsulin) or 0.4 g (T1 encapsulins) of PEG-8000 was added and incubated for 30 min on ice. The precipitate was collected using centrifugation (8000 rpm, 15 min, 4 °C) and the supernatant was removed. The pellet was suspended in 3 mL low salt buffer (20 mM Tris, 100 mM NaCl, pH 8) and filtered with a 0.2 μm syringe filter. The solution was subjected to size exclusion chromatography using an ÄKTA Explorer 10 (GE Healthcare, Life Sciences) equipped with a HiPrep 16/60 Sephacryl S-500 h column with a flowrate of 1 ml min^−1^. Fractions were selected by fluorescence and concentrated and dialyzed using Amicon Ultra Filters with 100 kDA molecular weight cut-off (Milipore). Concentrate was suspended in 3 mL of 20 mM Tris, pH 8. The sample was loaded on a HiPrep DEAE FF 16/10 Ion Exchange column (GE Healthcare Life Sciences). Fluorescence was used to select fractions, followed by concentrating using Amicon Ultra Filters with 100 kDA molecular weight cut-off (Milipore). Concentration was determined using a Nanodrop ND-1000 instrument (PEQLab). His-tagged mNeonGreen was purified using Ni–NTA agarose resin (Qiagen) via a batch Ni–NTA affinity step, according to the supplier’s instructions. After elution, the samples were dialyzed and concentrated using Amicon Ultra filters with a 10 kDa molecular weight cut-off (Milipore). Samples were either used directly or stored at 4 °C.

### Negative stain transmission electron microscopy

All experiments were carried out at the HMS Electron Microscopy Facility using a Tecnai G2 Spirit BioTWIN electron microscope at 80 keV. Samples were diluted to 1 mg mL^-1^ using low salt buffer and adsorbed onto 200 mesh formvar carbon-coated gold grids (EMS). To increase hydrophilicity, grids were glow-discharged using a 100× glow-discharge unit (EMS) for 10 s at 25 mA before applying the sample. Grids were placed onto a 5 μL droplet of sample for 1 min; excess liquid was blotted away using Whatman #1 filter paper, washed in H_2_O and placed on a 10 μL droplet of fresh uranyl formate (0.75% in H_2_O) for 30 s. After removal of any excess liquid the samples were ready for imaging.

### Polyacrylamide gel electrophoresis (PAGE)

All reagents and equipment for gel analysis were purchased from *ThermoFisher Scientific*. All gels were imaged using a *ChemiDoc-MP (Biorad)*.

#### SDS-PAGE

Protein samples were mixed with 3× loading buffer containing β-mercaptoethanol, boiled for 15 min and spun down. Samples were run on Novex 14% Tris–Glycine PAGE gels for 2 h at 100 V. Gels were stained in Coomassie Brilliant Blue, rinsed with water, and destained in a hot 20% acetic acid solution. Densitometry was performed using Image lab 5.2.1 (Biorad).

#### Native PAGE

Novex 3–12% Bis–Tris NativePAGE gels were used and run according to the manufacturer’s instructions and recommended buffers.

### Bulk fluorescence measurements

Samples were diluted to 15 μg mL^−1^ in low salt buffer and supplemented with 1 mg mL^−1^ BSA. 40 μL of the mixture was put on a black low flange flat 384 well plate (Corning) in triplicates. Fluorescence of mNeonGreen was measured with a Synergy H1 plate reader (BioTek) using the monochromator at Ex: 500 nm, Em: 530 nm, 16 nm band width, gain: 100. During measurements, the temperature was set to 20 °C. The % loading was calculated by using the concentration of mNeonGreen in the sample, determined using a standard curve (Supplementary Information Fig. S8) of purified mNeonGreen as shown in Eq. (1).

1 $$\%=\frac{[mNeonGreen]}{\left[Protein\right]-[mNeonGreen]}*100\%$$

### Single particle fluorescence measurements

#### Sample preparation

Custom made flow chambers were made by sandwiching two pieces of double-sided tape (Scotch, 3 M) 5 mm apart between a pre-cleaned microscope glass slide (25 × 75 × 1 mm, VWR) and coverslip (25 × 25 mm, VWR). 20 μL of a 200× or 1000× diluted sample (T1 or T3, respectively) were incubated for 5 min in the flow chambers. Flow chambers were washed with low salt buffer prior and after sample loading and sealed with epoxy glue.

#### Imaging

All images were collected with a wide field Nikon Ti motorized inverted microscope with a Plan Apo 60× NA 1.4 lens objective and the Perfect Focus System for maintenance of focus over time. mNeonGreen fluorescence was excited with a Prior LumenPro fluorescence light source with HQ 480/40, 505lp and ET 535/50 filters (Chroma) for excitation, dichroic and emission, respectively. Images were acquired with a Hamamatsu ORCA-R2 cooled CCD camera controlled with MetaMorph 7.2 software. A 5 × 5 grid of images was obtained with an exposure of 2.5 s (T1) or 0.75 s (T3). Movement of the slide was controlled by Prior Proscan III linear-encoded motorized stage.

#### Image analysis

ImageJ software was used. A selection of 512 × 512 pixels in the center of each image was used for further analysis. All images were subjected to flat field correction using the CIDRE plugin^29^. Individual spots were selected and measured using a custom script. Spots were identified based on local maxima; intensity of a spot was determined using the GaussFit OnSpot plugin.

#### Data analysis

Origin 2015 was used. Intensity bin size for each sample was calculated according to Eq. (2), Freedman-Diaconis rule. Mean and Mean Absolute Deviation (Eq. 3) were determined using Origin 2015.

2 $$Bin\;size=2* \frac{IQR}{\sqrt[3]{} n}$$

3 $$MAD= median (|{X}_{i}-median (X)|)$$

### Computational modeling

#### Structure preparation

Proteins structures were obtained from PDB, T1: 3DKT2, T3: 4PT2. One subunit was selected and subjected to Rosetta Relax^30–33^, in the case of T3, two neighboring subunits were used. Subsequently, the peptide was placed linearly over the binding pocket. To make sure the docking protocol worked optimally, the complex of protein and peptide was prepacked^34^.

#### Peptide docking

FlexPepDock protocol was used as described by Raveh et al.^26,27^. At least 15,000 structures were created. To make sure only reasonable peptide angles were used, all possible rotamers during docking were calculated using PSIPRED^35–37^. As scoring function for the docking process, the minusRama score, was used, as defined by Zheng et al.^38^.

#### Analysis

First, all the structures were sorted based on their score. The top 500 by lowest score were taken and clustered with a RMSD of 3 Å. The lowest energy structure in the largest cluster was chosen to be the representative.

#### Point mutations

The best scoring model of T3 was used to introduce the mutations to. After mutating, 100 FlexPepDock refinement runs were performed for each mutation. The lowest energy conformation of each mutation was used for analysis. To calculate the energy difference from the mutation, the native structure was subtracted from the mutated one. Energy of each mutation was normalized to the energy of the native residue at that position and represented in a heatmap.

### Protein identification

Excised SDS-PAGE gel bands were processed at the Harvard Medical School Taplin Mass Spectrometry Facility. Excised bands were divided into small ca. 1 mm^3^ pieces. They were washed and treated with acetonitrile for 10 min. Following speed-vac drying of the gel pieces, rehydration was carried out with 50 mM NH_4_HCO_3_ solution containing 12.5 ng/ml trypsin (Promega, Madison, WI) at 4 °C. Excess trypsin solution was removed after 45 min and replaced with 50 mM NH_4_HCO_3_ solution. After overnight incubation at 37 °C, peptides were extracted by first removing the NH_4_HCO_3_ solution, followed by a wash step using 50% acetonitrile/1% formic acid. Then, extracts were dried for 1 h. Samples were reconstituted in 5–10 mL water containing 2.5% acetonitrile and 0.1% formic acid. Samples were analyzed using a 2.6 μm C_18_ column (inner diameter: 100 μm, length: 30 cm). Samples were eluted using a linear gradient of solvent B (97.5% acetonitrile, 0.1% formic acid). Tandem MS/MS analysis was carried out using an LTQ Orbitrap Velos Pro ion-trap mass spectrometer (Thermo Fisher Scientific, Waltham, MA). Protein identities were determined using Sequest (Thermo Fisher Scientific, Waltham, MA).

## Supplementary Information

Supplementary Information.

## Data Availability

All protocols, materials and constructs used in this study are available upon request from the corresponding author.
