# Supplementary material for: Exploring targeting peptide-shell interactions in encapsulin nanocompartments
Source: Sci Rep. 2021 Mar 2;11:4951. doi: 10.1038/s41598-021-84329-z (PMC7925596; doi:10.1038/s41598-021-84329-z)
Supplement: Supplementary file 1 — Supplementary Information. [file 41598_2021_84329_MOESM1_ESM.pdf]

# Supplementary Information

# Exploring targeting peptide-shell interactions in encapsulin nanocompartments

Wiggert J. Altenburg[1,2], Nathan Rollins[1,2], Pamela A. Silver[1,2] and Tobias W. Giessen[1,2,3,4*]

[1] Department of Systems Biology, Harvard Medical School, Boston, MA 02115, USA

[2] Wyss Institute for Biologically Inspired Engineering at Harvard, Boston, MA 02115, USA

[3] Department of Biomedical Engineering, University of Michigan, Ann Arbor, MI 48109, USA

[4] Department of Biological Chemistry, University of Michigan Medical School, Ann Arbor, MI 48109, USA

[*] Corresponding author: tgiessen@umich.edu

## Table of Contents

Additional modeling data ........ 2
Encapsulin purification ........ 4
Additional PAGE gels ........ 5
Standard curve for mNeonGreen fluorescence ........ 8

## Additional modeling data

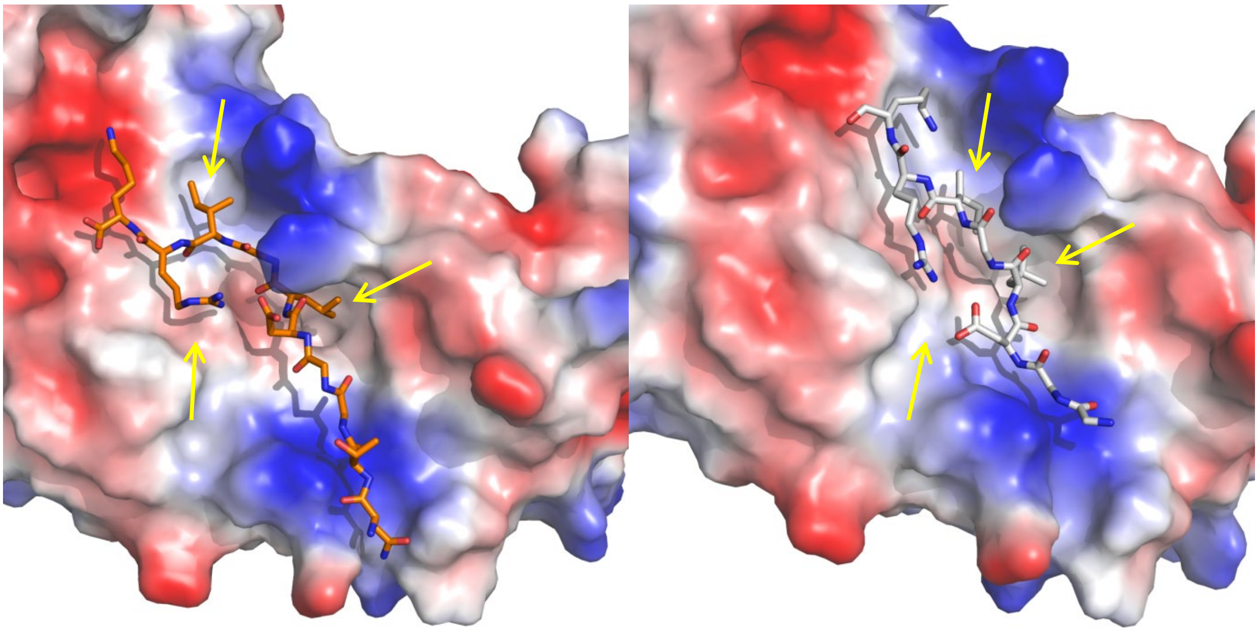

**Figure S1. Comparison of best calculated binding interaction with x-ray structure of the T1 *T. maritima* encapsulin.** Left: Modeled structure. Right: X-ray structure (3DKT). Yellow arrows indicate key interactions. The computed binding mode does recapitulate the intra-peptide interaction between the penultimate arginine and the glutamate residue. PyMOL Molecular Graphics System, Version 1.8.2.0. was used to create the surface representations and atomic structures shown (http://www.pymol.org).

**Table S1. Metrics from Rosetta FlexPepDock for all the best scoring models.**

| **T1** | **minusRama score** | **Total Score** | **Rama** | **Omega** | **Peptide_score** | **Reweighted Score** |
|---|---|---|---|---|---|---|
| **Native** | -392.478 | -351.805 | 49.777 | -9.104 | 1.163 | -376.499 |
| **Consensus** | -390.189 | -350.946 | 49.720 | -10.477 | 1.076 | -374.954 |
| **Control** | -385.955 | -350.310 | 46.990 | -11.345 | -5.238 | -371.539 |
| **T3** | **minusRama score** | **Total Score** | **Rama** | **Omega** | **Peptide_score** | **Reweighted Score** |
| **Native** | 453.953 | 847.084 | 350.944 | 42.187 | -2.876 | 814.846 |
| **Consensus** | 474.573 | 867.827 | 351.049 | 42.205 | 3.733 | 856.684 |
| **Control** | 472.279 | 860.525 | 347.959 | 40.287 | -8.078 | 846.304 |

**Table S2. Raw data of the refinement runs for all point mutations in the native T3 targeting peptide.** This data is the foundation for the heat map shown in Figure 5. Energy contribution per residue is shown. The native sequence is highlighted in green.

| | 1 | 2 | 3 | 4 | 5 | 6 | 7 | 8 | 9 | 10 | 11 |
|---|---|---|---|---|---|---|---|---|---|---|---|
| A | 9.00 | -5.687 | 0.393 | -8.615 | 8.634 | 2.104 | -0.067 | -2.201 | 3.648 | 4.026 | 2.251 |
| C | 1.03 | -3.067 | -1.510 | -0.667 | -1.732 | 7.448 | -2.501 | 4.176 | 8.195 | 7.967 | 5.197 |
| D | 2.12 | -2.307 | -3.070 | 4.042 | 2.089 | 6.025 | 1.808 | -0.864 | 5.901 | 13.349 | 4.608 |
| E | 4.92 | -6.151 | 5.078 | 5.711 | -1.074 | 3.821 | 1.299 | 11.709 | 7.545 | 0.526 | 10.921 |
| F | 4.70 | -4.372 | -2.383 | -4.634 | 2.303 | 6.912 | 0.838 | 7.945 | 4.464 | 12.993 | 7.549 |
| G | 6.25 | -7.751 | -2.214 | 0.279 | 0.000 | 2.168 | 6.301 | 4.574 | 9.587 | 0.000 | 0.000 |
| H | 2.28 | -1.153 | -1.653 | 3.886 | 1.688 | 7.914 | 3.703 | 0.831 | 2.917 | 7.085 | 5.354 |
| I | -3.07 | -5.181 | -1.324 | 1.449 | 14.795 | 9.626 | -1.544 | -2.580 | 13.152 | 4.411 | 5.753 |
| K | 6.04 | -1.421 | -3.641 | 18.086 | 1.855 | 4.781 | 0.776 | 13.067 | 3.342 | 7.677 | 12.966 |
| L | 9.87 | 0.000 | -4.615 | 3.193 | 2.317 | 4.614 | 0.000 | -2.736 | 2.670 | 0.938 | 12.804 |
| M | 2.95 | 1.248 | -5.732 | 11.514 | 5.453 | 9.013 | 0.665 | 8.821 | 13.152 | 14.061 | 9.778 |
| N | 8.17 | 1.496 | 0.321 | -2.467 | 6.212 | 14.943 | 2.012 | 5.703 | 17.454 | 4.549 | 8.845 |
| P | 0.00 | -0.438 | -6.061 | 6.233 | 1.138 | -2.478 | 1.418 | 2.011 | 9.698 | 4.570 | 6.653 |
| Q | 1.25 | -0.865 | -1.218 | 4.390 | 7.646 | 12.256 | 1.448 | -1.886 | 6.102 | 10.228 | 1.968 |
| R | 3.07 | -6.252 | -0.412 | 7.060 | 6.573 | 11.221 | 5.422 | 0.000 | 0.000 | 12.170 | 8.665 |
| S | 7.77 | -4.507 | -1.528 | -2.801 | 4.094 | 0.000 | 2.947 | -4.878 | 0.717 | 7.585 | 7.652 |
| T | 0.77 | -10.787 | 0.000 | 1.307 | -0.535 | 8.625 | 1.123 | 2.271 | 1.943 | 7.520 | 9.431 |
| V | -0.37 | -7.070 | -2.549 | 0.000 | 1.566 | -0.509 | -0.349 | -3.682 | 10.030 | 11.862 | 11.276 |
| W | 8.76 | 2.547 | -2.870 | 3.513 | 9.463 | 3.904 | 1.276 | 1.716 | 5.007 | 7.415 | 2.353 |
| Y | 7.00 | 4.163 | -6.568 | -0.270 | 8.564 | 12.077 | -0.067 | 4.952 | 0.423 | 7.613 | 8.251 |

## Encapsulin purification

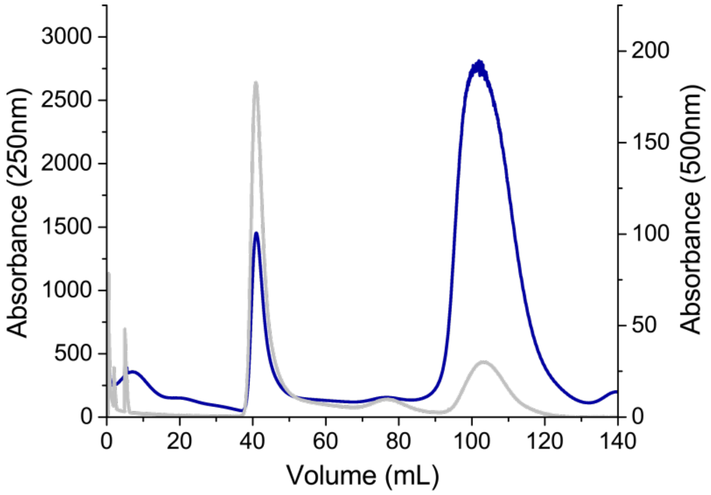


**Figure S2. First encapsulin purification step.** Sample chromatogram of a T1 sample + mNeonGreenTP (native) purification using a HiPrep 16/60 Sephacryl S-500 HR size exclusion column. Blue: Absorption at 280 nm. Gray: Absorption at 500 nm.

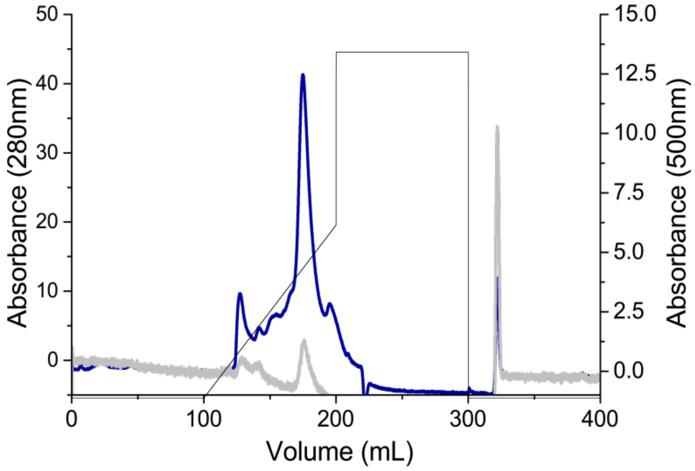


**Figure S3. Second encapsulin purification step.** Sample chromatogram of a T1 sample + mNeonGreenTP (native) purification using a HiPrep DEAE FF 16/10 Ion Exchange column. Blue: Absorption at 280 nm. Gray: Absorption at 500 nm.

## Additional PAGE gels

T1:

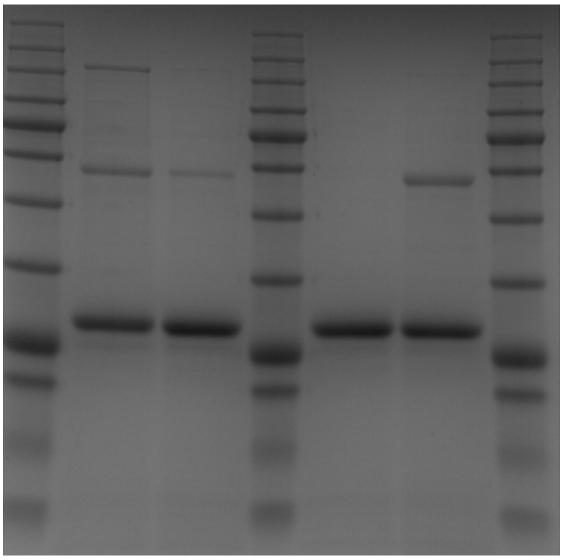

**Figure S4. Sample SDS-PAGE gel of purified T1 encapsulins.** Left to right: MW Marker, T1 Enc no cargo, native TP, MW Marker, consensus TP, control TP. Full gel image of the lanes shown in Figure 3 top row. As outlined in the main text, cargo and the T1 capsid protein could not be separated on the gel but were confirmed via in-gel tryptic digest followed by mass spectrometry analysis of the resulting peptide fragments.

T3:

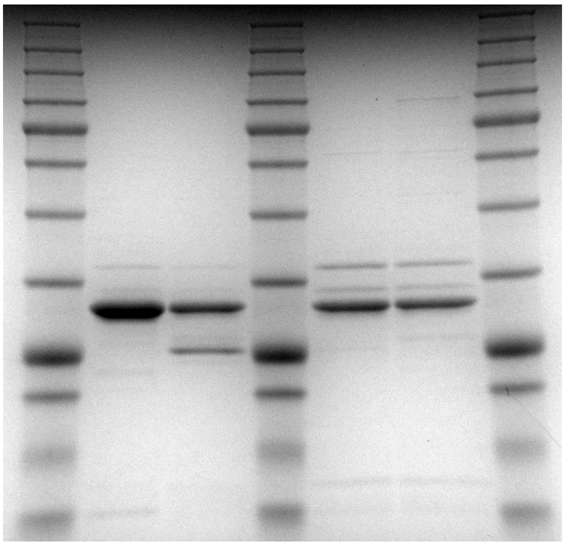

**Figure S5. Sample SDS-PAGE gel of purified T3 encapsulins.** Left to right: MW Marker, T3 Enc no cargo, native TP, MW Marker, consensus TP, control TP. Full gel image of the lanes shown in Figure 3 bottom row.

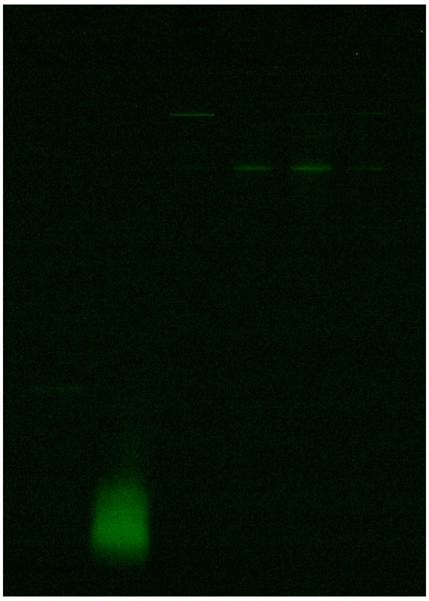

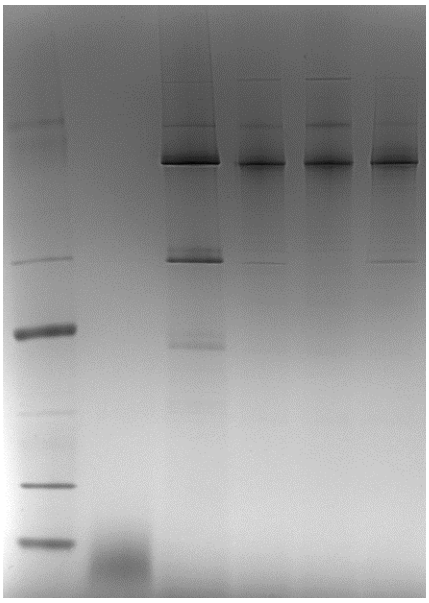

**Figure S6. Sample Native PAGE gel for T1 encapsulins.** Top: Fluorescence image of the gel at 500 nm, Bottom: Coomassie stained gel. Samples Left to right: Ladder (NativeMark Unstained Protein Standard, Life Technologies), mNeonGreen, no cargo, consensus TP, native TP, control TP.

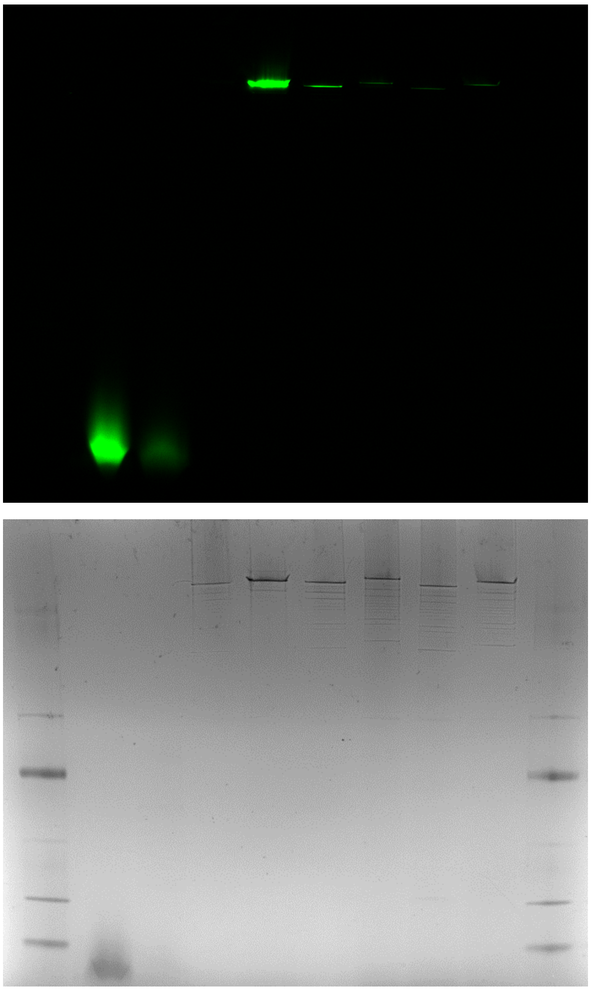

**Figure S7. Sample Native PAGE gel for T3 encapsulins.** Fluorescence image of the gel at 500 nm, Bottom: Coomassie stained gel. Samples Left to right: Ladder (NativeMark Unstained Protein Standard, Life Technologies), mNeonGreen, 1:2 dilution of mNeonGreen, no cargo, native TP, consensus TP, 1:2 dilution of consensus TP, 1:3 dilution of consensus TP, control TP, ladder.

## Standard curve for mNeonGreen fluorescence

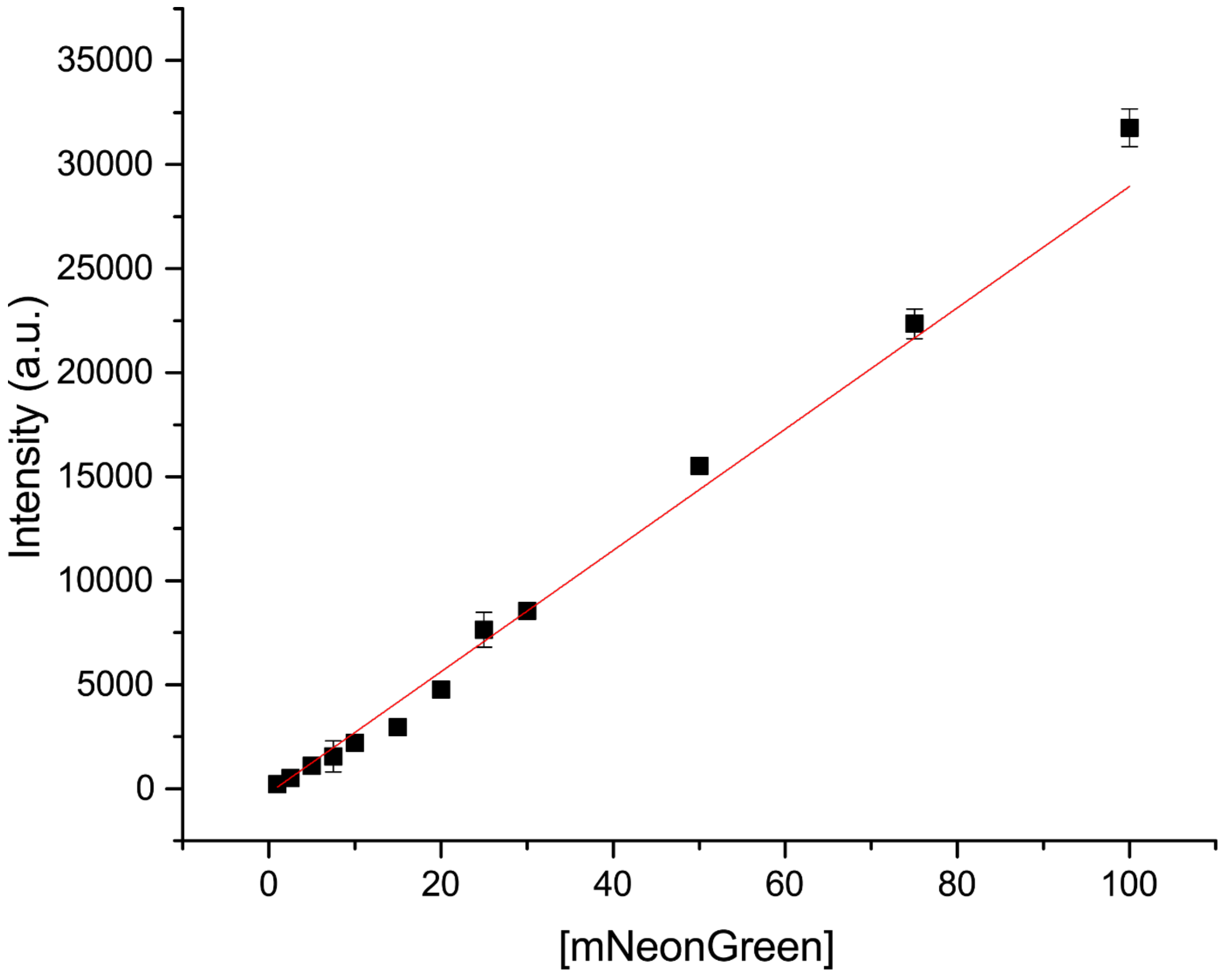


**Figure S8. mNeonGreen fluorescence standard curve.** Measured intensity vs mNeonGreen concentration in nM. Fit: x = (y+215.1)/291.7.

.
